# Flavored Cannabis Product Use Among Adolescents in California

**DOI:** 10.5888/pcd18.210026

**Published:** 2021-06-03

**Authors:** Miranda Werts, Janelle Urata, Shannon Lea Watkins, Benjamin W. Chaffee

**Affiliations:** 1 University of California, San Francisco, California; 2 University of Iowa, Iowa City, Iowa

## Abstract

Given the well-documented role of flavors in encouraging tobacco use among adolescents and diversity of the cannabis market, we describe flavored cannabis product use, both smoked and aerosolized (“vaped”), among a sample of adolescents. We surveyed 1,423 students in 8 Northern and Central California public high schools (2019–2020) to record flavored tobacco and cannabis use. Among past 30-day cannabis users, use of flavored cannabis, most often fruit-flavored, was common for smoked (48.1%) and vaped (58.0%) products. Given that youth-appealing flavors may contribute to underage cannabis use, emerging cannabis control policies should consider lessons from tobacco control to prevent youth cannabis use.

**Summary**

**What is already known on this topic?**

Cannabis use among youth is increasing. Flavors in tobacco products are associated with youth appeal and experimentation.

**What is added by this report?**

In this sample of California adolescents, approximately half of those who recently used smoked cannabis and the majority who used aerosolized (“vaped”) cannabis reported using a flavored product, most often fruit or dessert, in the past month.

**What are the implications for public health practice?**

Within an expanding legal cannabis marketplace, this report highlights a potential role for restrictions on marketing cannabis flavors and flavored additives, including flavored cigar and vaporizer products often used to consume cannabis, to discourage cannabis use among adolescents.

## Objective

Cannabis (marijuana) is frequently used by US high school students, with almost 36% of 12th-grade students reporting past-year use (1). Cannabis products, including oil vaporizers, cannabis flower, and blunt wraps, are advertised with flavor, taste, and food descriptors (eg, “sweet,” “fruit,” “lemon haze”) (2). Among adolescent tobacco users, use of flavored products, particularly for electronic cigarettes (e-cigarettes), is common (3) and reported as a motivation for tobacco use (4). Little work has documented patterns of flavored cannabis product use (5), particularly among youth. Here, we describe flavor-related behaviors among adolescent tobacco and cannabis users to help inform potential cannabis control policy.

## Methods

An in-person, school-based survey was administered to grade 9 and 10 students (N = 1,423) in 8 public high schools in Northern and Central California in 2019–2020, as described elsewhere (6). Schools were in municipalities with fewer than 50,000 residents and in counties of population density less than 1,000 people per square mile. The survey assessed both past 30-day and ever use of tobacco and cannabis, perceptions of tobacco products, socio-environmental variables, and health conditions. Participating students received a $10 gift card to an online retailer and each school received $300. Parents provided written consent and students assented. The institutional review board at the University of California, San Francisco, approved all study procedures.

Our cross-sectional analysis examined baseline data collected from March 2019 to February 2020. Ever and past 30-day use prevalence of cigarettes, cigars, e-cigarettes, moist snuff, and cannabis were calculated for the entire sample based on separate survey items for each product that included brief product descriptions and photographs. Students who reported past 30-day cannabis use were asked to report how they consumed cannabis from a list of methods, categorized as smoked (joints, blunts, spliffs, pipes, bong, or moke), vaped (vaporized or dabbed flower, bud, oil, wax, extract, liquid, or concentrate), or other (eg, edible or tincture).

Separately for each tobacco product and for smoked and vaped cannabis routes of administration, past 30-day users were asked which flavors they had used in the past 30 days (the options were not mutually exclusive), later grouped as “flavored,” “not flavored or tobacco flavor only,” and “don’t know.” Among cannabis past 30-day users (smoked and/or vaped), we also calculated the unweighted frequency of reporting each specific flavor (eg, fruit, alcoholic drink). This descriptive analysis does not include hypothesis tests and was conducted using Stata 16.0 (StataCorp LLC).

## Results

Approximately half of the sample identified as female (53.4%), as Hispanic or Latino (53.1%), and/or as qualified for free or reduced-price school lunches (54.2%). More than one-third of adolescents had ever used cannabis (37.7%) or tobacco (44.6%, any tobacco product, including e-cigarettes). Prevalence of past 30-day use was 21.0% for cannabis (any form) and 23.2% for tobacco (any product, including e-cigarettes) (Table 1).

**Table 1:** Flavored Product Use Among Past 30-Day Users, by Product (N = 1,423), California High School Students, 2019–2020^a^

Product	Cigarettes	Moist Snuff	Cigars	E-Cigarettes	Smoked^b^ Cannabis	Vaped^c^ Cannabis
**Past 30-day product use, among total sample**	32 (2.2)	20 (1.4)	36 (2.5)	302 (21.2)	160 (11.3)	139 (9.8)
**Flavored use, among past 30-day users^d^**						
Flavored^e^	14 (43.8)	13 (68.4)	29 (80.6)	225 (75.8)	76 (48.1)	80 (58.0)
No flavor or tobacco flavor^f^	15 (46.9)	6 (31.6)	7 (19.4)	27 (9.1)	39 (24.7)	24 (17.4)
Don't know	3 (9.4)	0 (0.0)	0 (0.0)	45 (15.2)	43 (27.2)	34 (24.6)
Missing data^g^	0	1	0	5	2	1

a Values are number (unweighted percentage).

b Includes joints, blunts, spliffs, pipes, bong, or moke (cannabis and tobacco waterpipe).

c Includes vaped or dabbed flower, bud, oil, wax, extract, liquid, or concentrate.

d Percentages may not add to 100% because of rounding.

e Includes only mint or menthol for cigarettes; users of other products selected ≥1 option from a list of flavor categories (eg, fruit, mint, dessert, other [Table 2]).

f Used only nonflavored or tobacco-flavored products in the past 30 days.

g Missing values excluded from denominator for calculating percentages.

Flavored product use was common for tobacco and cannabis. Most past 30-day users of moist snuff (68.4%), cigars (80.6%), and e-cigarettes (75.8%) used flavored products, and 43.8% of past 30-day cigarette users reported smoking mint or menthol cigarettes (Table 1). Among smoked cannabis users (n = 160), 48.1% reported using a flavored smoked cannabis product, and 58.0% among vaped cannabis users (n = 139) reported using a flavored aerosolized cannabis product (Table 1).

Among responses indicating a flavor, fruit was the most common category for both smoked (30.4%) and vaped (39.1%) cannabis (Table 2). Candy, dessert, or other sweet flavors was the next most common flavor category, whereas use of other flavors such as alcoholic drink, mint, and menthol was infrequently reported (Table 2).

**Table 2:** Types of Flavors Used Among Past 30-Day Cannabis Users, California High School Students, 2019–2020

Flavor	No. (Unweighted Percentage)^a^
**Smoked cannabis (n = 160)**	
No flavor^b^	56 (35.4)
Fruit	48 (30.4)
Candy, dessert, or sweet	38 (24.1)
Alcoholic drink	9 (5.7)
Spice or cinnamon	8 (5.1)
Mint (not menthol)	6 (3.8)
Menthol (cool or frost)	4 (2.5)
Nonalcoholic drink	0 (0.0)
Other flavor	8 (5.1)
Don’t know^c^	48 (30.4)
Missing data^d^	2
**Vaped cannabis (n = 139)**	
Fruit	54 (39.1)
No flavor^b^	43 (31.2)
Candy, dessert, or sweet	38 (27.5)
Alcoholic drink	8 (5.8)
Mint (not menthol)	7 (5.1)
Menthol (cool or frost)	5 (3.6)
Spice or cinnamon	5 (3.6)
Nonalcoholic drink	2 (1.4)
Other flavor	2 (1.4)
Don’t know^c^	41 (29.7)
Missing data^d^	1

a Not mutually exclusive: respondents could select ≥1 flavor option, including “don’t know” and “no flavor.”

b Not mutually exclusive: includes those who selected “no flavor” even if another flavor was also selected.

c Not mutually exclusive: includes those who selected “don’t know” even if another flavor was also selected.

d Missing values excluded from denominator for calculating percentages.

## Discussion

These results show that a substantial proportion of adolescent cannabis users are choosing flavored cannabis products, including both combustible and aerosolized products. This finding illuminates a potential health concern, as adolescents associate flavors in tobacco products with less perceived harm and have greater interest in experimentation with flavored tobacco compared with unflavored tobacco products (6,7). Flavored cannabis might have similar effects. Many US states permit or have decriminalized medical and/or recreational cannabis use (8), corresponding with rising levels of cannabis use among youth and adults (9). As the commercial cannabis industry offers an expanding array of products promoted as flavored, potential restrictions on flavored cannabis may prove an important component of limiting youth appeal.

What is considered flavored cannabis may reflect a variety of use patterns. Smoked cannabis may be consumed by using flavored rolling papers, as a blunt within a flavored tobacco cigar (10,11), or with cannabis flower that was marketed with flavor descriptors (2). Questionnaire items did not differentiate between flavors as additives, wrappers, or taste characteristics of cannabis strains; future work should consider these differences. Using flavored cigar products for cannabis consumption is particularly popular among cannabis users (5,10), suggesting that potential coordination of tobacco and cannabis flavor restriction policies may beneficially reduce both tobacco and cannabis use among youths.

This study had limitations. Results from this sample of California schools in smaller towns may not generalize to other geographic contexts; e-cigarette and cannabis use were higher than from earlier statewide estimates (12). The sample size precluded precise estimates of flavor use prevalence. As markets co-evolve, future work should examine associations between flavored cannabis and flavored tobacco use, including their co-administration and use patterns longitudinally.

The observed levels of flavored cannabis and flavored tobacco use in this study underscore the relevance of flavors in both tobacco and cannabis control policy. Restrictions that prohibit sales of any characterizing flavors, such as recent local and state restrictions on the sale of flavored tobacco products (eg, New York City [13] and San Francisco [14]), could help address rising adolescent interest in new tobacco products and cannabis use.
